# Supplementary material for: Coffea canephora: Heterotic Crosses Indicated by Molecular Approach
Source: Plants (Basel). 2022 Nov 9;11(22):3023. doi: 10.3390/plants11223023 (PMC9692650; doi:10.3390/plants11223023)
Supplement: Supplementary file 1 [file plants-11-03023-s001.zip › plants-1956484-supplementary.pdf]

Coffea canephora: heterotic crosses indicated by molecular approach

Modes for *Coffea canephora* and Coffea arabica

K=1 - 20/20, Mean(LnProb) = -1043.365, Mean(similarity score) = 1.000

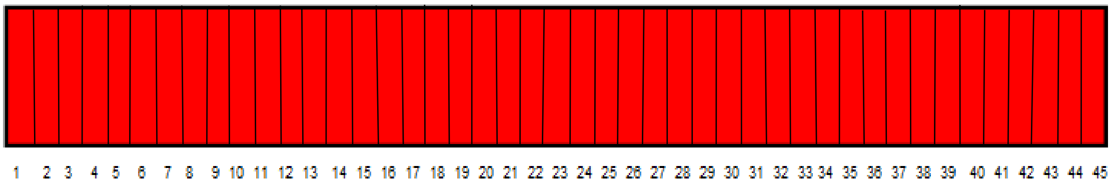

K=2 - 20/20, Mean(LnProb) = -998.710, Mean(similarity score) = 0.994

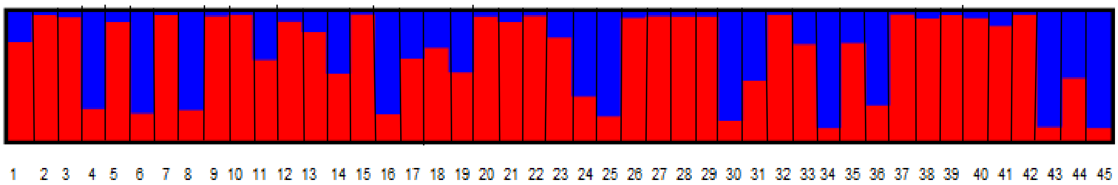

K=3 - 20/20, Mean(LnProb) = -965.675, Mean(similarity score) = 0.997

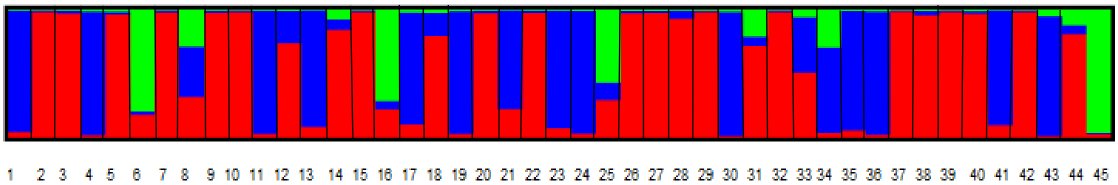

K=4 - 20/20, Mean(LnProb) = -974.990, Mean(similarity score) = 0.994

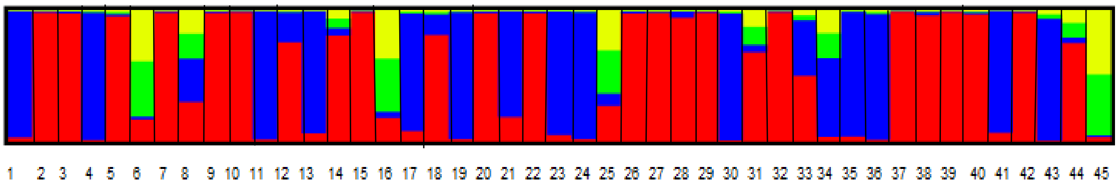

K=5 - 20/20, Mean(LnProb) = -975.055, Mean(similarity score) = 0.994

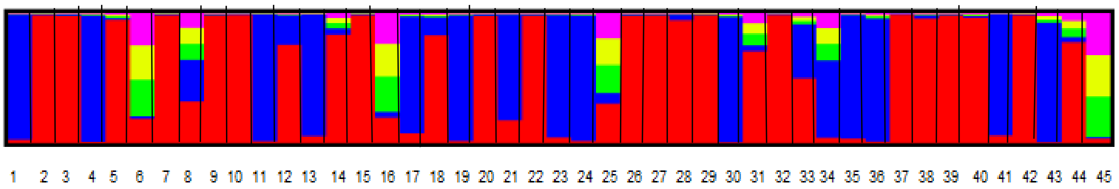

K=6 - 16/20, Mean(LnProb) = -975.805, Mean(similarity score) = 0.993

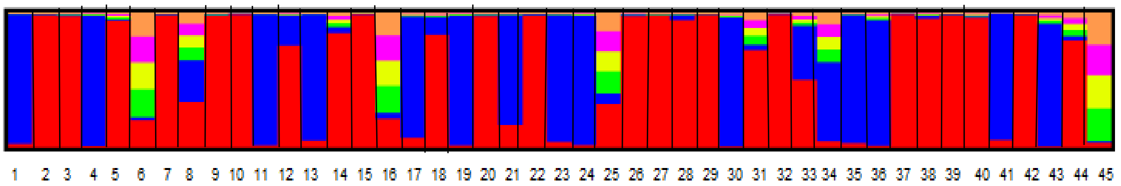

K=7 - 20/20, Mean(LnProb) = -976.870, Mean(similarity score) = 0.994

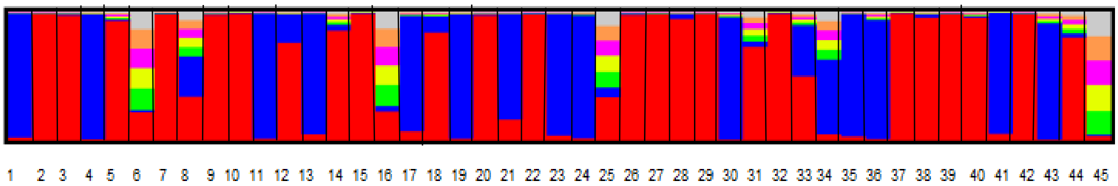

K=8 - 20/20, Mean(LnProb) = -978.250, Mean(similarity score) = 0.994

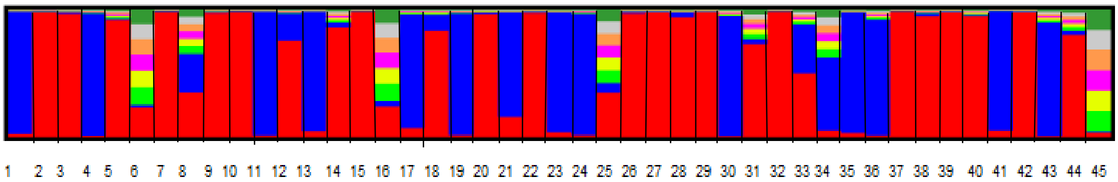

K=9 - 20/20, Mean(LnProb) = -979.380, Mean(similarity score) = 0.995

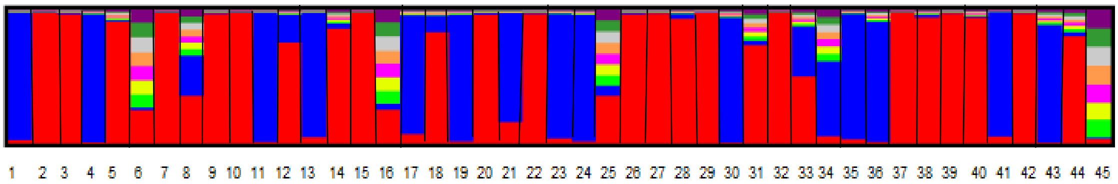

K=10 - 20/20, Mean(LnProb) = -980.900, Mean(similarity score) = 0.995

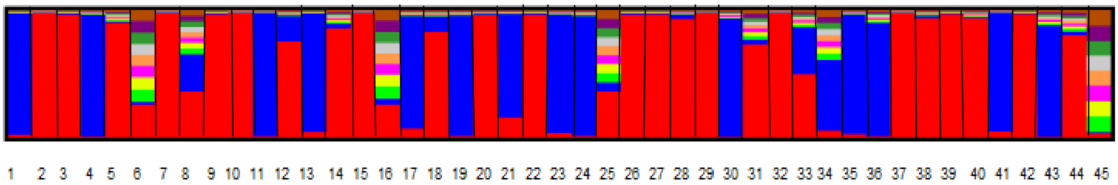

Division of runs by mode:

K=120/20

K=220/20

K=320/20

K=420/20

K=520/20

K=620/20

K=720/20

K=820/20

K=920/20

K=120/20

Figure S1. Structure cluster graphs of 44 *C. canephora* and one genotype of *C. arabica* in Brazil with minor and major modes for the data. The runs were performed for K = 1 to 10 with 20 replicates and division for runs by mode is provided below the graphs. In the graph, each horizontal bar represents an individual palm tree, and the proportion of each color in the bar corresponds to the proportion of

the individual genotype assigned to a given cluster. Codes corresponding to their identification. Individuals labels: 1 = AP; 2 = Ouro negro 2; 3 = 18; 4 = Ouro negro 1; 5 = Graudão HP; 6 = Peneirão; 7 = Ouro Negro; 8 = Z18; 9 = Clementino; 10 = Beira Rio 8; 11 = VerdimD; 12 = Z39; 13 = Bamburral; 14 = P2; 15 = Imbigudinho; 16 = AT; 17 = Emcapa 153; 18 = Bicudo; 19 = Alecrim; 20 = Z38; 21 = 122; 22 = Sementes; 23 = Valcir P; 24 = Clone 1; 25 = Z40; 26 = CH1; 27 = Emcapa 143; 28 = VerdimR; 29 = Tardio C; 30 = Clone 2; 31 = 700; 32 = Z29; 33 = Emcapa 02; 34 = Pirata; 35 = A1; 36 = Z36; 37 = Z37; 38 = B01; 39 = Tardio V; 40 = Z21; 41 = Z35; 42 = LB1; 43 = L80; 44 = P1; 45 = Arábica.

Table S1. The average in percentage of estimated membership coefficients of each individual to the inferred K cluster for 45 C. canephora genotypes and one C. arabica genotype

Ind.	Identificação	Cluster 1 – Red	Cluster 2 - Dark blue	Cluster 3 – Light green
1	AP	4.32%	95.03%	0.65%
2	Ouro negro 2	98.20%	1.18%	0.62%
3	18	97.18%	1.98%	0.84%
4	Ouro negro 1	1.46%	97.15%	1.39%
5	Graudão HP	96.38%	1.93%	1.69%
6	Peneirão	17.54%	3.05%	79.41%
7	Ouro Negro	97.94%	1.46%	0.60%
8	Z18	31.81%	39.12%	29.07%

9	Clementino	97.83%	1.37%	0.81%
10	Beira Rio 8	98.19%	1.31%	0.50%
11	Verdim D	2.44%	96.86%	0.70%
12	Z39	73.84%	25.26%	0.90%
13	Bamburral	8.31%	91.09%	0.60%
14	P2	84.08%	8.39%	7.53%
15	Imbigudinho	98.27%	1.20%	0.53%
16	AT	21.66%	6.85%	71.50%
17	Emcapa 153	10.00%	87.87%	2.13%
18	Bicudo	79.35%	18.30%	2.35%
19	Alecrim	2.57%	96.27%	1.16%
20	Z38	97.38%	1.82%	0.80%
21	122	21.99%	77.41%	0.60%
22	Sementes	97.52%	1.68%	0.80%
23	Valcir P	7.13%	92.17%	0.70%
24	Clone 1	2.69%	96.31%	1.00%
25	Z40	29.25%	13.54%	57.21%
26	CH1	97.24%	1.87%	0.88%
27	Emcapa 143	97.67%	1.33%	1.00%
28	Verdim R	93.12%	6.19%	0.69%
29	Tardio C	98.20%	1.00%	0.80%

30	Clone 2	1.10%	97.23%	1.67%
31	700	71.43%	7.35%	21.22%
32	Z29	98.27%	1.19%	0.53%
33	Emcapa 02	50.68%	43.46%	5.86%
34	Pirata	3.54%	66.98%	29.48%
35	A1	5.40%	93.71%	0.89%
36	Z36	1.90%	96.52%	1.58%
37	Z37	98.41%	1.09%	0.50%
38	B01	95.57%	3.47%	0.96%
39	Tardio V	98.30%	1.10%	0.60%
40	Z21	96.87%	1.93%	1.21%
41	Z35	9.51%	90.01%	0.48%
42	LB1	98.18%	1.22%	0.60%
43	L80	1.02%	93.87%	5.11%
44	P1	80.94%	6.87%	12.19%
45	Arábica	2.20%	1.30%	96.50%

% Individual Q values from the file: 1656000816/ind
